# Diet behaviour among young people in transition to adulthood (18–25 year olds): a mixed method study

**DOI:** 10.1080/21642850.2014.931232

**Published:** 2014-08-28

**Authors:** Amudha S. Poobalan, Lorna S. Aucott, Amanda Clarke, William Cairns S. Smith

**Affiliations:** ^a^ Public Health Nutrition Research Group, Division of Applied Health Sciences, University of Aberdeen, Polwarth Building, Foresterhill, AberdeenAB25 2ZD, UK; ^b^ School of Health, Community and Education Studies, Northumbria University, Coach Lane Campus, Benton, Newcastle upon TyneNE7 7XA, UK

**Keywords:** young adults, 18–25 year olds, diet behaviour, mixed methods study, obesity

## Abstract

*Background* **:** Young people (18–25 years) during the adolescence/adulthood transition are vulnerable to weight gain and notoriously hard to reach. Despite increased levels of overweight/obesity in this age group, diet behaviour, a major contributor to obesity, is poorly understood. The purpose of this study was to explore diet behaviour among 18–25 year olds with influential factors including attitudes, motivators and barriers. *Methods*: An explanatory mixed method study design, based on health Behaviour Change Theories was used. Those at University/college and in the community, including those Not in Education, Employment or Training (NEET) were included. An initial quantitative questionnaire survey underpinned by the Theory of Planned Behaviour and Social Cognitive Theory was conducted and the results from this were incorporated into the qualitative phase. Seven focus groups were conducted among similar young people, varying in education and socioeconomic status. Exploratory univariate analysis was followed by multi-staged modelling to analyse the quantitative data. ‘Framework Analysis’ was used to analyse the focus groups. *Results*: 1313 questionnaires were analysed. Self-reported overweight/obesity prevalence was 22%, increasing with age, particularly in males. Based on the survey, 40% of young people reported eating an adequate amount of fruits and vegetables and 59% eating regular meals, but 32% reported unhealthy snacking. Based on the statistical modelling, positive attitudes towards diet and high intention (89%), did not translate into healthy diet behaviour. From the focus group discussions, the main motivators for diet behaviour were ‘self-appearance’ and having ‘variety of food’. There were mixed opinions on ‘cost’ of food and ‘taste’. *Conclusion*: Elements deemed really important to young people have been identified. This mixed method study is the largest in this vulnerable and neglected group covering a wide spectrum of the community. It provides evidence base to inform tailored interventions for a healthy diet within this age group.

## 1. Background

Young adults (18–25 years) in transition from adolescence to adulthood, addressed as ‘emerging adults’, begin independent living, embark on higher education/employment, start living with partners or get married and/or become parents themselves and potentially are vulnerable to weight gain (Anderson, Shapiro, & Lundgren, 2003; Burke, Beilin, Dunbar, & Kevan, 2004; Graham & Jones, 2002). Individual health behavioural patterns developed during this transition often persist into later life (Parcel, Muraskin, & Endert, 1988) influencing their own health, that of their partners and/or children. Between the years 1991 to 2001, the greatest increase in obesity (BMI > 30) was found amongst young adults between the ages of 18–29 years (7.1–14%) (Huang et al., 2003; Mokdad et al., 2003). The promotion and sustained behavioural change is best achieved from a targeted approach, by identifying transition points in the life course when individuals are at a higher risk of weight gain (Leermakers, Anglin, & Wing, 1998; NICE, 2007). In spite of this recognition, young adults between 18 and 25 years of age are a neglected age group compared with children or middle-aged adults and are hard to reach (Howarth & Street, 2000; The Prince's Trust, 2004).

Among the reasons for weight gain for 18–25 year olds are dietary pattern changes (breakfast skipping, eating outside home) and increased social activities contributing to lifestyle changes (Huffman & West, 2007; Niemeier, Raynor, Lloyd-Richardson, Rogers, & Wing, 2006; Sheehan, DuBrava, DeChello, & Fang, 2003). Although they have positive attitudes towards dietary advice, these are not usually reflected in their behaviour (Mullaney, Corish, & Loxley, 2008). Once obese, young people not only suffer from premature medical consequences but also face discrimination in employment settings, health care facilities and educational institutions (Puhl & Heuer, 2009).

Before developing an intervention, it is crucial to explore as many contributory factors as possible for this specific target population. For obesity prevention, this should include dietary behaviour as well as physical activity. Previous studies addressing diet and exercise that included young people using behavioural theories (Bozionelos & Bennett, 1999; Caperchione, Duncan, Mummery, Steele, & Schofield, 2008; Wallace, Buckworth, Kirby, & Sherman, 2000) have been conducted either in a wider age group, focused specifically on university students or were based only on quantitative study methodology. This study is one of the first to explore attitudes, intentions and diet behaviour along with related lifestyle factors in this vulnerable age group. Further a mixed method study design was used based on health behaviour change theory incorporating both quantitative and qualitative results.

## 2. Methods

A sequential explanatory mixed method design was used to understand diet behaviour and related lifestyle factors amongst 18–25 year olds living in the Grampian area of North-East of Scotland through a questionnaire survey and focus groups. Explanatory mixed method design (Creswell & Clark, 2007) is a two phased study, with initial quantitative data collection followed by qualitative methods. The qualitative aspect follows on from the quantitative data and is used to explain or expand on the initial quantitative results.

### 2.1. Data collection methods

#### 2.1.1. Questionnaire design and coding

Guided by a National Health Service (NHS) Grampian steering group, a questionnaire was designed to collect quantitative data (questionnaire available on request). It was based on the Theory of Planned Behaviour (TPB) (Nutbeam & Harris, 1999) and Social Cognitive Theory (SCT) (Bandura, 2004), both commonly used for health behaviour change and previously validated questionnaires. Attitudes, subjective norm and intention constructs were drawn from TPB and the barriers and facilitators were drawn from SCT. Consequently, the questionnaire included demographic factors along with self-reported height and weight; diet behaviours (fruit and vegetable consumption, meal pattern and snacking); diet attitudes, their subjective norm which is a person's belief about what is expected of him/her, their intention to eat healthily and barriers/facilitators for eating a healthy diet.

##### 2.1.1.1. Diet behaviour

To represent a healthy diet, the National guidelines for eating ‘5 a day’ was specified in the questionnaire and participants were asked the number of times they ate fruits and vegetables as two separate questions. These were combined to get an overall measure of their *Fruit and vegetable consumption* behaviour and then determined as either ‘adequate’ if a mixture of fruits and vegetables were eaten six times a day or ‘not adequate’ otherwise. To get the *Regular Meal pattern behaviour*, three questions were asked about the frequency of eating breakfast, lunch and dinner in a week. These were grouped into ‘regular breakfast’ or ‘regular lunch’ or ‘regular dinner’. Then those who regularly had breakfast, lunch and dinner either every day or 3–6 times a week were coded as having a ‘regular meal pattern’ and the rest as ‘not regular’. For *Snacking* behaviour, participants were asked the number of times they snacked each day, in addition to any main meals. The snacks included were initially defined as chocolate bars/sweets, crisps/savoury snacks, sugary fizzy drinks, diet/sugar-free fizzy drinks and fruit juice/diluting juice, each with an option of ‘none’ up to having ‘more than 3’. These were combined into an overall snack count and regrouped into tertiles representing ‘low snacking’ if they had three or less snacks a day, ‘medium snacking’ if they had 4–5 snacks a day and ‘high snacking’ if they had more than six snacks a day.

##### 2.1.1.2. Diet attitudes

Attitudes towards a healthy diet using the surrogate eating ‘5 a day’ every day was assessed using four validated concepts – For me, eating ‘5 a day’ every day would be: ‘unpleasant/pleasant, ‘worthless/worthwhile’, ‘unhealthy/healthy’ and ‘stupid/clever’. These were assessed by a 5-point scale 1 (disagree) up to 5 (agree). A question on diet intention asked about the intention of young adults to eat ‘5 a day’. This was coded from 1 (disagree) up to 5 (agree), but for the modelling this was dichotomised such that 1 and 2 were grouped together as ‘disagree’ and compared to 4 and 5 ‘agree’. It was felt that those chose 3 were either neutral about intention or genuinely did not know or did not sufficiently care hence were excluded. These approaches were approved by the health psychologists (Araújo-Soares, 2006).

##### 2.1.1.3. Facilitators and barriers for a healthy diet

Two questions asked about what facilitators would encourage healthier eating. The responses from the first ‘what would encourage them to eat more healthy food’ were condensed into three facilitator sub-groups (health, appearance and subjective norm) while the second ‘changes that would help them to eat more healthily’ had four sub-groups (provision for more opportunities, more information, more support and more choices).

Similarly questions assessing barriers that is factors that would prevent them from having a healthy diet, were reduced to a lack of ‘time’, ‘access’ to healthy food, ‘money’, an inability to ‘cook’, lack of ‘support’ and not ‘enjoying’ healthy food.

#### 2.1.2. Sample recruitment for the quantitative survey

Recruitment of the sample was possible only by approaching educational institutions, since direct access to young adults was not permitted due to corporate policies. Consequently, the questionnaire was sent electronically via institutes to all university/college students in the Grampian area in 2008. They were asked to complete the questionnaire if they were between 18 and 25 years of age (those not in this range were filtered out). To capture young adults not in education, employment or training (NEET), hard copies were sent to co-ordinators of the NEET groups in the Grampian area to be completed by participants at their groups meetings. To capture those at work and young adults who may not attend the NEET group sessions, a postal hard copy of the questionnaire was sent to a 2% random sample of 18–25 year olds (*n* = 1800) in the community using the community health index (CHI), a computer-based population index used by NHS Scotland.

#### 2.1.3. Focus groups

A purposive sampling method was used informed by the quantitative survey results (age, level of education, employment status) with the intention of obtaining a balance in terms of socio-economic groups. Using the university website, young adults between the ages of 18 and 25 years were invited to participate in focus group discussions using a ‘pop up’ advert. An institutional e-mail with an information letter was also sent to all the students. All the NEET groups and other youth groups/clubs in Grampian area were again approached through the group co-coordinators and local media (radio). Seven focus groups resulted with a total of 26 participants from the same target population as the quantitative survey. Focus groups gather participants' attitudes, feelings, beliefs, experiences and reactions in a collective way, not feasible using other methods such as observation, one-to-one interviewing or questionnaire surveys (Gibbs, 1997). A topic guide, based on issues identified from the survey and grounded in TPB and SCT, facilitated discussion. Participants were also encouraged to discuss relevant issues of concern to them, ensuring an inductive approach. Questions addressed in the focus group discussions related to actual diet behaviour; the importance and perceived relevance of healthy eating at this stage of life and in the future; positive and negative outcome expectations; perceived and actual barriers to healthy eating; and, finally, factors that might facilitate and encourage healthy eating. The focus group data collection was terminated after obtaining saturated data from a wide range of relevant groups. Written informed consent was obtained from participants at the time of the focus groups ensuring anonymity and confidentiality. Ethical approval was obtained from NHS Grampian for the quantitative study and from university ethics committee for the qualitative study.

### 2.2. Data analysis methods

For the questionnaire survey all analyses were performed using SPSS version 21 and all assumptions were checked. Initially, univariate analyses were conducted identifying significant variables (using the most appropriate χ^2^ tests) then a multi-staged model was developed to explore diet behaviours with respect to the theoretical mediating variables. Focus groups discussions were analysed using ‘framework analysis’.

#### 2.2.1. Exploratory univariate analysis

Obesity prevalence and frequencies of all the behavioural theory constructs, except perceived behavioural control were assessed with demographic factors. The associations between the TPB constructs (attitudes and subjective norm) were first assessed with behavioural intention and then with each of the diet behaviours. Similarly the SCT constructs (barriers and facilitators) were analysed with demographics and the diet behaviours.

#### 2.2.2. Multistage modelling

After identifying significant variables from the univariate analyses detailed above, a strategic methodology was developed for modelling, using a blocked approach, executed in three stages. Initially, *behavioural intention* (intend to eat healthily: yes or no) was modelled against demographic/risk factors and each TPB construct (attitudes and subjective norm (SN)) using logistic regression. Significant variables from these were considered together and again reduced to include only significant variables. Secondly, each *Diet behaviour* was separately modelled against (1) demographics, (2) constructs of TPB, (3) intention (4) barriers and (5) facilitators. Finally, a model utilising all variables significant from these latter stages and the *Intention* model was developed to predict each diet behaviour. Given that *Fruit and Vegetable* and *Regular meal pattern* behaviours were reduced to binary outcomes, logistic regression was again used. However, *Snacking* behaviour had three categories and required nominal regression. All the modelling was conducted using forward selection to identify the significant variables using the normal default entry significance levels. These final model(s) provided the most important associations for the diet behaviours considered here.

#### 2.2.3. Framework analysis

Framework analysis was used to analyse focus group data in a systematic way (Ritchie, Spencer, & O'Connor, 2005). Framework analysis uses a thematic framework to classify and organise data according to a priori themes and concepts and also emergent categories from the data. It allows transparent data management and comparison of data between groups. As each group was analysed, themes were added and amended until an agreed framework of themes was developed. Data were therefore explored within a common framework that was both grounded in the theory and informed by participants' views and experiences.

After analysing the quantitative and qualitative data separately using their respective appropriate analytical approaches, a ‘side-by-side comparison’, method was used. This enabled the comparisons and synthesis of the results from both quantitative and qualitative components (Creswell & Clark, 2011).

## 3. Results of questionnaire survey

### 3.1. Exploratory and univariate analysis

#### 3.1.1. Obesity and diet behaviour

There were 1313 completed questionnaires analysed (1029 from university/colleges and 284 from the community). The sample characteristics are given in Table 1. Self-reported overweight/obesity was 22%, increasing with age (21.8% in 18–19 year olds and 31.9% in 23+). Males were more frequently obese than females (9.4% obese in males compared to 6.8% obese in females). In terms of diet behaviour, 40% of young adults consumed adequate amounts of fruits and vegetables (having a mixture of fruits and vegetables 6 times a day); 59% had regular meals (three meals every day or 3–6 times a week) and a third (32%) had more than six unhealthy snacks a day.

**Table 1.** Demographic details of the 1313 respondents.

Demographics	Frequency	Percentages
*Gender*		
Male	353	26.9
Female	929	70.8
Missing	31	2.4
*Age*		
18–19 years	471	35.9
20–22 years	592	45.1
23+	250	19.0
*Employment and study status*		
Student	856	65.2
Employed	164	12.5
Student and employed	238	18.1
Others^a^	54	4.1
Missing	1	0.1
*Subject of study (if student)*		
Arts	284	21.6
Health	174	13.3
Science	453	34.5
Others	184	14.0
Missing (probably not students)	218	16.6
*Year of study*		
Foundation (including HND/HNC)	610	46.5
Undergraduate	419	31.9
Postgraduate	116	8.8
Missing (probably not students)	168	12.8
*Time spent in Aberdeen*		
Less than a year	239	18.2
1–3 years	335	25.5
4 years or more	735	56.0
Missing	4	0.3
*Living arrangements*		
Living alone all of the time	145	11.0
Living alone Mon–Fri	46	3.5
Living with others	1115	84.9
Missing	7	0.5
*Smoking*		
Non-smoker	960	73.1
Less than or equal to 5 a day	149	11.3
More than 5 a day	200	15.2
Missing	4	0.3
*Alcohol consumption*		
Low	872	66.4
Medium	125	9.5
High	284	21.6
Missing	32	2.4

Notes: HND: Higher national diploma; HNC: higher national certificate.

^a^Unemployed, long-term sick and others.

#### 3.1.2. Attitudes, subjective norm and intentions towards ‘eating 5 a day’

Young adults had positive attitudes towards eating ‘5 a day’ considering it to be healthy (85%), worthwhile (72%), pleasant (56%) and clever (74%), Table 2. When asked if expectations from parents and friends (subjective norm) would make them eat more fruits and vegetables, only 17% thought that it was important and 83% did not answer this statement. The participants gave positive attitudes and good intentions (89%) to eat adequate fruits and vegetables. Their actual fruit and vegetable eating ‘behaviour’ was only adequate in 40%.

**Table 2.** Frequencies of the health outcomes and the mediating theoretical constructs from theory of planned behaviour.

Health outcomes and theoretical constructs	Frequency	Percentages
*BMI categories (self-reported height and weight)*		
Underweight	379	28.9
Acceptable weight	549	41.8
Overweight	199	15.2
Obese	89	6.8
*Diet behaviour*		
* Overall fruit and vegetable consumption*		
Adequate fruit and vegetable	522	39.8
Inadequate fruit and vegetable	696	53.0
*Meal pattern*		
Regular meals everyday	772	58.8
Irregular meals everyday	541	41.2
*Snacking*		
Low (None to 3)	437	33.3
Medium (4 or 5)	378	28.8
High (6 or more)	415	31.6
*Attitude to eating ‘5 a day’*		
1 (unpleasant)	44	3.4
2	56	4.3
3	207	15.8
4	263	20.0
5 (pleasant)	737	56.1
1 (worthless)	19	1.4
2	30	2.3
3	102	7.8
4	209	15.9
5 (worthwhile)	941	71.7
1 (unhealthy)	9	0.7
2	10	0.8
3	35	2.7
4	136	10.4
5 (healthy)	1111	84.6
1 (stupid)	16	1.2
2	13	1.0
3	102	7.8
4	198	15.1
5 (clever)	966	73.6
*Diet – subjective norm (eating 5 a day)*		
Important	94	7.2
Not very important	129	9.8
Missing	1090	83.0
*Diet intention (eating 5 a day)*		
1 (disagree)	22	1.7
2	32	2.4
3	81	6.2
4	198	15.1
5 (agree)	970	73.9

Note: Percentages do not add up to 100% due to missing values.

#### 3.1.3. Associations between the individual constructs and demographic factors

Health outcomes/constructs had similar univariate associations with age as with levels of education. Those in the younger age group (18–19 year olds) were heavy snackers (more than 6 a day) but no age associations with fruit and vegetable consumption or meal eating pattern were found. Foundation level (basic level) students, likely to be younger, had significantly more irregular meal eating patterns and were heavy snackers. The postgraduate students, likely to be older, felt that eating ‘5 a day’ was pleasant and worthwhile and had strong intentions to have a healthy diet. Pleasing others with their diet behaviour was important for foundation level students (37.3% compared to 11.1% in post graduates). The intention to eat healthily was stronger in 20–22 year olds (77.3%) compared to the 18–19 year olds (69.8%).

With respect to gender, there was no difference in the amounts of fruits and vegetables eaten, but males were more likely to be heavy snackers and had irregular meal eating patterns. Females thought that eating ‘5 a day’ was pleasant, worthwhile, healthy and clever and they had strong intentions to have a healthy diet. Compared with students studying ‘other’ subjects, those studying health-related subjects were more likely to have regular meals, eat adequate amounts of fruits and vegetables which they found to be ‘pleasant’ and was a ‘clever’ thing to do. Those in education had more positive attitudes towards eating ‘5 a day’ and had stronger intentions to eat healthily compared to those who were ill/unemployed. Students also had regular meal eating patterns with low snacking levels compared to employed young adults. Heavy smokers did not eat adequate amount of fruits and vegetables, tended to snack heavily and had irregular meal eating patterns. Moderate smokers (smoked 1–5 cigarettes a day) thought that eating ‘5 a day’ to be pleasant, worthwhile, healthy and clever compared to non-smokers. Young adults living with other people and those with low alcohol consumption also tended to have regular meal eating patterns.

#### 3.1.4. Association between the individual constructs

Young adults with positive attitudes towards eating ‘5 a day’ and wanting to please others had strong intentions to eat healthily. When it came to diet behaviour, those who ate adequate amounts of fruits and vegetables or had regular meal eating patterns or low snacking levels generally had positive attitudes towards diet. Despite this, there was no significant association between snacking and healthy/unhealthy attitudes. While young adults who wanted to please others had a strong intention to eat ‘5 a day’, this was not associated with any of the diet behaviours. Those with strong intentions to have a healthy diet consumed adequate amounts of fruits and vegetables and had regular meal eating pattern but had varied snacking behaviour.

#### 3.1.5. Association between diet behaviours and body mass index

There was no significant association between BMI and fruit and vegetable consumption in young adults (*p* = 0.586). However, the level of obesity was significantly higher for those with irregular meal eating patterns (9.5% compared to 5.9%; *p* < 0.001). There was a significant association between meal patterns and snacking behaviour (*p* < 0.001), where those with higher levels of snacking had irregular meal eating patterns. In turn, there was an increasing trend in obesity with increasing snacking albeit non-significant, data not shown.

### 3.2. Multistage modelling

#### 3.2.1. Behavioural intention model

Using separate logistic regression models on the demographic, attitude and subjective norm blocks, indicated that ‘intention to eat 5 a day’ (y/n) (Table 3) was associated with ‘gender’, ‘employment status’, ‘year of study’ and various attitudes (Eating ‘5 a day’ being ‘unpleasant/pleasant’, ‘stupid/clever’ and ‘unhealthy/healthy’). When considered together, ‘gender’, ‘employment status’ and just two attitudes (‘unpleasant/pleasant’ and ‘unhealthy/healthy’) remained significant explaining around 50% of the overall variation in this intention construct (using Nagelkerke *R* ^2^ = 0.542, commonly used to give a sense of fit for logistic regression models).

**Table 3.** Relationship between the Intention and demographics, attitudes and subjective norm. Intention was assessed by the statement ‘I intend to Eat “5 a day” every day’. Agree (ref) vs disagree by attitudes + subjective norm + intention + demographics.

Demographics *n* = 1162, ‘Agree’ = 1111 vs ‘Disagree’ = 51, Nag *R*^2^ = .209		Attitudes towards eating ‘5 a day’ *n* = 1203, ‘Agree’ = 1151 vs ‘Disagree’ = 52, Nag *R*^2^ = .511		Subjective norm *n* = 208, ‘Agree’ = 202 vs ‘Disagree’ = 6, Not significant
Age group		Unpleasant/pleasant	‡*	Diet subjective norm
Gender	‡*	Stupid/clever	‡	
Employment status	‡*	Unhealthy/healthy	‡*	
Year of study	‡	Worthless/worthwhile		
Subject				
BMI category				
Alcohol category				
Quantity of cigarettes				
Living arrangement				
Combined diet intention model, *n* = 1184, ‘Agree’ = 1133 vs ‘Disagree’ = 51, Nag *R*^2^ = 0.547				

Notes: PA, Physical activity; SN, Subjective norm; Nag *R* ^2^, Nagelkerke *R* squared – pseudo measure of model fit. *n* represents the sample size that was included in each model.

‡Significant at *p* < 0.05 in each block model.

*Significant at *p* < 0.05 in the combined model.

#### 3.2.2. Fruit and vegetable consumption behaviour

As a surrogate for healthy eating, ‘5 a day’ was reduced to a dichotomous variable; those who ‘eat adequate fruit and vegetables’ compared to those who ‘do not eat adequate fruit and vegetables’. Significant variables identified (Table 4(a)) were ‘subject’ studied and ‘smoking status’ from the demographic block; Eating ‘5 a day’ being ‘unpleasant/pleasant’, ‘stupid/clever’ and ‘unhealthy/healthy’ from the attitude block; ‘intention to eat 5 a day’ itself with the facilitators that healthy eating helped improve ‘appearance’ and was more likely if there was adequate ‘information’. Having no ‘cooking ability’, a lack of ‘time’, not ‘enjoying’ cooking and having insufficient ‘money’ were identified as significant barriers. The final model for *Fruit and vegetable consumption* behaviour used significant variables combined from the *Behavioural Intention* model and those identified above for this behaviour. Variables ‘age group’, ‘employment status’, ‘year of study’, ‘subject’ studied, an attitude (eating ‘5 a day’ is ‘unpleasant/pleasant’), a facilitator ‘appearance’ along with two barriers (insufficient ‘time’ and ‘money’) remained in this final model explaining around 20% of the variation (Nagelkerke *R* ^2^ = 0.210). Those eating sufficient fruit and vegetables tended to study science-related subjects, felt that eating ‘5 a day’ was pleasant and would enhance their appearance while, those not eating adequate amounts of fruit and vegetables were likely to find time and money to be barriers. See supplemental Table S1 for effect sizes.

**Table 4.** Relationship between the three diet behaviours with demographics, attitudes, subjective norm including the barriers and facilitators of healthy diet.

Block	(a) Fruit and vegetable consumption– logistic regression, adequate (ref.) vs not adequate		(b) Regular meal patterns – logistic regression, total amount– regular (ref), not regular		(c) Snacking – nominal regression, total snack categories – low (ref),med, high	
Demographics	*n* = 1146, Nag *R*^2^ = 0.031, Adequate = 48, not = 659		*n* = 1185, Nag *R*^2^ = 0.117, Regular = 718, not = 467		*n* = 1114, Nag *R*^2^ = 0.091, Low = 401, Mod = 347, High = 366	
Age group						†*
Gender	^‡^		‡		‡	†*
Employment status	‡		‡	† *	‡	†*
Year of study						
Subject		† *				
BMI category				† *		†
Alcohol category				† *		
Smoking status		†		† *		†*
Living arrangement						
Attitudes	*n* = 1212, Nag *R*^2^ = 0.145, Adequate = 521 vs not = 691		*n* = 1301, Nag *R*^2^ = 0.038, Regular = 768 vs not = 533		*n* = 1226, Nag *R*^2^ = 0.040, Low = 437, Mod = 376, High = 413	
Eating ‘5 a day’ is						
Unpleasant/pleasant	‡	† *	‡	† *	‡	†*
Stupid/clever						
Unhealthy/healthy	‡		‡		‡	
Worthless/worthwhile				†		
Subjective norm	*n* = 205, not significant		*n* = 223, not significant		*n* = 207, not significant	
Intention	*n* = 1142, Nag *R*^2^ = 0.008, Adequate = 508 vs not = 634		*n* = 1222, Nag *R*^2^ = 0.007, Regular = 740 vs not = 482		*n* = 1145, not significant, Low = 414, Mod = 351, High = 380	
You would like to eat ‘5 a day’		†		†		
Barriers	*n* = 1169, Nag *R*^2^ = 0.053, Adequate = 501 vs not = 668		*n* = 1260, Nag *R*^2^ = 0.030, Regular = 737 vs not = 523		*n* = 1182, Nag *R*^2^ = 0.031, Low = 421, Mod = 361, High = 400	
Cooking ability		†		†*		
Support						†*
Time		† *				
Enjoy		†		†		†
Access						
Money		† *		† *		
Facilitators	*n* = 1029, Nag *R*^2^ = 0.033, Adequate = 451 vs not = 575		*n* = 1107, Nag *R*^2^ = 0.011, Regular = 660 vs not = 447		*n* = 1083, Nag *R*^2^ = 0.009, Low = 390, Mod = 333, High = 360	
Health						†*
Appearance		† *		†		
Choices						
Information		†				
Opportunities						
Support						
Full model	*n* = 947, Nag *R*^2^ = 0.210, Adequate = 412 vs not = 535		*n* = 1155, Nag *R*^2^ = 0.139, Regular = 698 vs not = 457		*n* = 1025 , Nag *R*^2^ = 0.142, Low = 364, Mod = 317, High = 390	

Notes: Nag *R* ^2^: Nagelkerke *R* squared – pseudo measure of model fit. *n* represents the sample size that was included in each model.

‡Significant at *p* < 0.05 from *Intention* model.

†Significant at *p* < 0.05 from each Block.

*Significant at *p* < 0.05 from final behaviour model.

#### 3.2.3. Meal eating pattern behaviour

*Meal eating pattern*s (reduced to regular and irregular eating patterns) had independent significant associations with some demographic variables (‘employment status’, ‘BMI category’, ‘alcohol category’ and ‘smoking status’), an attitude (‘worthless/worthwhile’) and the intention to eat ‘5 a day’ but not with the subjective norm (Table 4(b)). In addition, barriers such as their inability to ‘cook’, not ‘enjoying’ healthy food and the lack of ‘money’ were significant with the facilitator ‘appearance’. These variables were incorporated with those significant from the *Intention* model. This indicated that the risk of having irregular meals was increased by being employed, either underweight or obese, a heavy smoker and/or drinker along with, finding eating ‘5 a day’ to be unpleasant or feeling they had insufficient money to eat healthily. Supplemental Table S2 gives the effect sizes which given the ‘fit’ of this model was also only around Nagelkerke *R* ^2^ = 14% so should be cautiously interpreted.

#### 3.2.4. Snacking behaviour

At a univariate level, *Snacking* behaviour determined by categories low, medium and high (Table 4(c)) was associated with some demographic variables (‘age group’, ‘gender’, ‘employment status’, ‘BMI categories’ and ‘smoking status’), an attitude (‘unpleasant/pleasant’), two barriers (lack of ‘support’ and not ‘enjoying’ healthy food) and one facilitator (wanting to be healthy). Neither intention nor the subjective norm was associated with snacking behaviour. When combined with the significant variables from the *Intention* model, the full snacking behaviour model (*R* ^2^ = 0.142, again not a good model for prediction) indicates that heavy snackers are more likely to be younger, male, employed and/or heavy smokers. They would also tend to find eating ‘5 a day’ to be unpleasant and/or that they lack support to eat healthily. The low-level snackers tended to have more positive attitudes about eating ‘5 a day’ and wanted to be healthy. Although the moderate snackers varied little from the low-level snackers, they had mixed feelings about the level of support they had for achieving a healthy diet and potentially smoked more (see supplemental Table S3).

## 4. Results of focus groups

Seven focus groups were conducted and the characteristics are presented in Table 5. Five themes and several subthemes were identified from a priori themes and emerging data following analysis: diet behaviour, influences on diet behaviour, knowledge and sources of information, attitudes and behaviour change.

**Table 5.** Characteristics of the focus groups.

Focus group	Code	Characteristics	No. of participants (M/F)	Mean age (range)
University (higher education)	T0	Older group	5 (1/4)	22 (20–24)
University (higher education)	C0	Younger group	8 (3/5)	19 (18–19)
College (further education)	V0	Working/training 1	2 (0/2)	21 (20–22)
College (further education)	M0	Working/training 2	2 (1/1)	20 (18–21)
Inner city (deprived areas)	H0	Young mothers	3 (0/3)	23 (21–24)
Inner city (deprived areas)	P0	Mixture of working/not working	4 (0/4)	19 (18–21)
Shire (rural area)	K0	Community youth group – not in education or employment	2 (1/1)	19 (18–19)

### 4.1. Diet behaviour

The older of the young adults (20+ age group), irrespective of being at university or college, were reasonably confident that their diet was generally healthy. However, younger adults (18–19 year olds) at university or college felt that, while they ate healthily before coming to university, their eating habits were now more irregular and reported skipping meals and having too many snacks and fizzy drinks. Young mothers believed that they did not have a chance to eat a proper meal while looking after their children, whilst those in paid employment described having more unhealthy takeaways and tended to snack more between meals. All groups admitted to going through phases of healthy eating:

I go through phases of eating healthy then pigging out, sort of thing. (College; 20–22 age group)

 … like I just eat healthy for a day or two. Just like eat fruit and dinna eat Takeaways and don't drink fizzy juice. Two days I'll last and I'll go back. (Young mothers; 21–24 age group)

University and college students felt that their unhealthy phase started when they moved away from home to live independently. In spite of the initial unhealthy phase, these student participants did return to relatively healthy eating (highlighted later under ‘reasons for changing unhealthy behaviour’) albeit with occasional lapses mainly during times of stress, exams and essay writing. All the young adults expressed a need to change their diet, frequently stating that always eating the same thing was ‘boring’ regardless of whether the food consumed was healthy or unhealthy and preferred variety in their food:

Nobody's going to want to eat spaghetti every night just because it is healthy. (University; 18–19 age group)

I couldn't eat the same thing … lot of folk that eat healthy …. only baguette … I couldn't eat one thing. (Shire – Not in education, employment or training-NEET; 18–19 age group)

### 4.2. Influences on diet behaviour

Childhood experiences and ‘mothers’ had a major positive influence on university and college students, even after a spell of unhealthy eating or times of stress. Some participants who were in college (still living with parents), and anticipated moving on to university, were anxious that they might become unhealthier after moving away, but were reasonably confident that they would still eat healthily. They acknowledged that their diet might not be as varied as home cooking. Others reported being introduced to a healthy diet by friends after they started living independently at university. It was felt that cheap health cafés providing healthy and affordable food on campus might have a positive influence on their diet. Young adults exposed to a healthy diet, who had tried to be healthy for a while, said they did so ‘*to feel better*’*,* ‘*look better*’ and to ‘*get into good habits*’:

OK, well may be my nails will look better now or my skin won't be as clogged up. Or I won't put on as much weight if I eat lower calorie food. (College; 20–22 age group)

Those in education felt that it improved their concentration, gave them more energy and helped them perform better, preventing the need to snack. Other reported influences included past health scares, to avoid weight gain/ ill health, avoid the feeling of being ‘*greasy*’ and ‘*dirty*’:

I think it's just generally better for you. It makes you feel better. I know that if I eat, if I eat an unhealthy meal you kind of feel greasy and horrible. (University; 20–24 age group)

While young adults in university and colleges were positively influenced by their parents/mothers and partners, those living in the inner city were not. They expressed feeling uncomfortable talking to their parents about their concerns such as their inability to cook, and some did not want to eat what their mothers cooked. Some participants from the inner city had no experience of eating a healthy diet as children or rarely ate fruit/vegetables. One of the strongest subthemes that came across in all groups as a negative influence was the lack of time or inability to plan/organise shopping and preparing meals, especially during stressful times, such as examinations/long working hours, looking after young children and lack of company. Other negative influences on diet behaviours were the close vicinity/easy access to fast foods, the unavailability of healthy food, the lack of cooking facilities (in university halls of residence) and being fussy about foods. Young adults from other countries felt that Britain was generally unhealthy and found it hard to buy non-fatty food and good bread:

In Sweden we eat so much healthy. I don't know what you do here but the food here is very unhealthy. Especially I live in halls. The food is really fat. (University; 18–19 age group)

Participants made several assumptions about food. There was a notion that eating ‘*vegetarian/organic food*’ and only those made from fresh ingredients were healthy, while eating products not grown locally/non-organic/tinned food /ready meals were unhealthy. It was felt that it was appropriate to have an unhealthy diet occasionally, if they generally had a reasonably healthy diet. Participants from one of the inner city groups also assumed that, as long as they exercised sufficiently, diet did not matter:

Not concerned about diet..no.. as long as you exercise…… enough stairs in Primark …  … .you burn your dinner off by the time you get down stairs. (Inner city-working; 18–21 age group)

The inner city groups thought that having a ‘smoothie’ was part of their ‘5 a day’. For a few young adults, their diet was based on strong values such as supporting the right industry (buying local produce) and seemed to be influenced by the subject studied at university (for example, conservation biology). ‘*Mood*’ seemed to affect their diet behaviour in general. Sometimes, unhealthy eating was described as a ‘spur of a moment decision’, taken without thinking or after drinking alcohol. There was no strong evidence that young people ate healthily to impress others. Although not explicit, university/college students seemed to believe that their behaviour reflected what their mothers taught them. One participant from the inner city expected to eat unhealthy when out with their siblings.

### 4.3. Knowledge and sources of information

In spite of some misconceptions about healthy diets (see section above), most of these young adults, irrespective of education or socioeconomic status, had a reasonable awareness of what constitutes a healthy and unhealthy diet. Participants were aware that eating fruits and vegetables (5 a day) benefited their general health. They recognised that eating in between meals and skipping meals was not good for them. Some believed that what they ate currently would have an affect later in life and that it would be hard to lose weight as they got older. Participants felt that there was enough information about healthy food, but recognised that there was a great deal of misinformation in the media and the internet (see *‘attitudes’* section) which could be misleading. Based on their knowledge, participants from the university group had tried to influence friends and flat mates' diets, but felt this was unsuccessful:

So he'll (flat mate) buy Weight Watchers ready meals, Weight Watchers yogurts, this and that I'm like … just make it yourself. It would be even better for you, and wouldn't cost you £4 a meal. … .And I'm like … , just watch me make soup. You chop up the vegetables put them in the pan, its fine. But he's like, no, no, no, its far too hard, these ones taste much better. He's just not really interested. (University; 20–24 age group)

We (participant and her friend) just have different diets. She has her ways. We're both quite set in our ways I think. We discuss it sometimes. But she likes what she likes, I like what I like. (University; 20–24 age group)

### 4.4. Attitudes

Participants felt the need for some ‘excitement’ from eating and that too much pressure to eat healthily could be counterproductive:

I would rather enjoy food than you know be skinny and be healthy. I think you need some fat in your diet and you need some excitement from eating as well or you just go off the whole experience. (University; 20–24 age group)

I think if you set out to do something (eat five a day) it makes it worse….because its like you've got to do it ..so I think that makes it harder. (Young mothers; 21–24 age group)

Taste was one of the major influences on food, but attitudes towards the taste of healthy and unhealthy food varied. There was general agreement that healthy food was tastier. Even those who did not eat fruit and vegetables did not give taste as a reason for not doing so. However, some thought that unhealthy food was more appetising. Participants from the inner city group said that they continued to eat unhealthy diets because these were cheaper and more accessible. This was in spite of disliking the taste and smell of some Fast Food.

Attitudes towards cooking were mixed. Some felt that cooking was relaxing and gave them the satisfaction of making something and knowing what had gone into it. Even during times of stress, university participants felt that they could spend time cooking healthily and were influenced in this respect by their upbringing and their mothers (as highlighted in the section on ‘*positive influences*’). Others disliked cooking; describing it as ‘a hassle’, requiring a lot of effort to prepare and cook. Participants from the inner city groups said they were unable to cook and were insecure about making a healthy meal. They agreed that being able to cook would encourage healthier eating.

When participants were asked if they were concerned about their diet/future health, across all the groups, ‘future health’ was not a main concern. The desire to look and feel ‘great’ was more important than any long-term health benefits:

Just like, well like, its not my first thought (health). Obviously that's the benefit of it but like if I start eating healthy it is like, ok, well maybe my nails will look better now or my skin won't be as clogged up. Or I won't put on as much weight if I eat lower calorie food. (College; 20–22 age group)

A few were fearful about their future health if they sustained an unhealthy diet but not sufficiently to promote healthy eating – as long as they did not put on weight.

Participants who thought that they ate healthily felt that their flatmates were unhealthy (because they did not cook with fresh ingredients) and those with an unhealthy diet also felt that their friends and families ate unhealthily. One participant said that she did not believe in counting calories or going to the gym for exercise. It was felt that there was enough information about health and ‘5 a day’, but the community group participants felt that health promotion messages were ‘useless’ and university students felt that information on healthy diets could be misleading:

(health messages) Heap of rubbish…. They don't look at what's advertised. They go for the food that they like. (Inner city-working; 18–21 age group)

To be honest…that (health messages) is a heap of crap (laughs), specially all that stupid milk shakes … what is going into them…don't understand. (Shire – NEET group; 18–19 age group)

### 4.5. Behavioural change

Being brought up in a healthy environment and being exposed to healthy food by friends when they first lived independently, helped participants to revert back to healthy eating behaviour. Although they were not getting fat, some stated that the lack of energy, feeling of disgust after eating unhealthy food, the recognition that it was not a viable way to continue and feeling guilty, helped them return to healthy eating behaviours. Young adults who ate relatively healthily showed some intention to keep up with a healthy diet. However, among participants who did not generally eat healthily, there was no strong intention to change their diet. Even those with good intentions were not very successful:

I always have good intentions about……but cake and things always gets me. Pizzas and stuff like that. (College; 20–22 age group)

Aye. I always say it (eat healthy) but never do it. …dinna ken. Its just. Dinna ken seem like a good idea….aye, yeah. But I never ever do it. Never. (Young mothers; 21–24 age group)

There was some evidence of perceived control over their diet among young adults. They were capable of saying ‘no’ to unhealthy food, were confident that they would eat reasonably healthily if they had to live on their own and could lose weight if they wanted to. When asked if anything would motivate them to consider changing their diet to being healthy, support from partners and family was discussed as one of the main motivators. Those who had the support in the past, succeeded in achieving their goal, while those who did not, had given up.

## 5. Discussion

This study explored the diet behaviour and influencing factors in this vulnerable and hard to reach age group. Included were, not only 18–25 year olds from university and colleges but also those working and not in education, employment or training (NEET groups). The mixed method study design identified factors affecting behaviour and unravelled details of these and other factors affecting behaviour through interactive focus groups discussion. This study showed that only around 40% ate adequate amounts of fruits and vegetables, 59% had three meals a day regularly, but around 60% of young people had more than four unhealthy snacks a day, consisting of chocolates, crisps and fizzy juices.

The model for the *Fruit and vegetable consumption* had the best fit compared to *Meal eating patterns* and *Snacking.* However*,* only regular meal patterns behaviour was significantly related to lower BMI. While regular meal eating patterns were also associated with lower levels of snacking, the latter was not additionally informative with respect to BMI. One-third of young people in this study regularly did not have breakfast compared to 8% and 3% skipping lunch and dinner, respectively. Fast food consumption and breakfast skipping have been seen to increase during the transition to adulthood and both of these behaviours are associated with weight gain (Niemeier et al., 2006). Previous studies in children have also shown that those who skipped breakfast had higher energy intake from snacks higher in fat (Gordon-Larsen, Adair, Nelson, & Popkin, 2004) probably due to hunger later in the day. This irregular eating and high snacking amongst young adults could be due to increased independence along with the added responsibility for obtaining food and its preparation. It is worth noting that the levels of obesity were higher amongst post-graduates and the older age group (23+), despite this group apparently choosing the healthy options: eating adequate amounts of fruits and vegetables, having positive attitudes and intentions towards healthy eating, having more regular meals and less snacking. Weight gains could be due to decreased level of physical activity as they get older (Poobalan, Aucott, Clarke, & Smith, 2012) perhaps in combination with a delayed impact of their higher levels of snacking and more negative attitudes when they were younger (18–19 years old). When it comes to employment status, it is interesting to note that obesity levels were lower in the employed group despite their irregular eating and increased snacking. Whether those working have higher levels of physical activity during work could not be determined, but would be worth investigating. In addition, there were higher levels of obesity among students who were also employed. This could possibly be because they were not sufficiently organised to prepare meals and perhaps had enough money to buy economical, energy-dense, readymade foods which are easily accessible and thus seen as an easy option.

Despite good intentions to eat adequate amounts of fruits and vegetables, young adults were unable to translate these into actual behaviour. While gender, employment status and positive attitudes explained 54% of the diet intention, translation of intention into behaviour was poor; intention even by itself only explained 1% of the variation in each of the *Fruit and vegetable consumption* behaviour and *Meal eating pattern* behaviour models and was not significant for *Snacking* behaviour (Table 4).

The qualitative study further revealed that diet behaviour varied among young adults. Although, participants were seen to be reasonably knowledgeable about the constituents of a healthy diet and the consequences of an unhealthy diet, there were some misconceptions (food should be organic, fresh or freshly prepared) and the belief that ‘healthy’ meant expensive. Diet behaviour was strongly influenced by parental/childhood experiences. Regardless of diet quality, this group stated that they needed ‘variety’ in their food but were driven on a daily basis by various stresses (such as examinations, lack of time, mood) and their organising skills or lack of, during those times. Consequently, there was evidence of going through ‘healthy eating phases’ with relapses when these factors came into play. Those who had gone through a healthy phase admitted to seeing benefits of their healthy diet. The first year of independent living at university and/or when first earning had a major influence on their unhealthy lifestyle, however, by around 20–21 years of age, there was a realisation that continuing with such an unhealthy diet is not viable and could motivate a positive change in diet.

The major motivators to either eating healthily or getting back to healthy eating seemed to be to ‘*look better*’ and ‘*feel great*’ now, rather than being concerned about future health, although this was of some concern they experienced occasionally. From the survey, ‘appearance’ was the main motivator for fruit and vegetable consumption and more regular eating, but for *Snacking* ‘health’ was more important than ‘appearance’. Focus group discussions revealed mixed opinions about the ‘cost’ and ‘taste’ of food. Young adults generally were not prepared to invest time and energy into cooking healthily but looked for easy options, even if they knew this was unhealthy. Support from family/friends and partners, along with skills to cook healthy food, were identified as possible motivators for changing. Understanding these varied perceptions among young adults would help in developing tailored interventions.

The major strength of the present study is that it captured a vulnerable age group (18–25 year olds) using a wide sample including not only students but also those who worked and those not in education, employment or training. The dynamic and interactive focus group discussions helped explain the in-depth meaning of the constructs, providing a better understanding of specific elements relevant for young people.

However, there are several limitations that need acknowledging. Although the data could represent typical Caucasian young adults in a similar transition phase, this data collection was restricted to particular part of Scotland and thus will be limited when extrapolated to young adults from other cultures especially with respect to the facilitators and barriers. In spite of the efforts made to recruit young adults from the community, either working full time or Not in Employment, Education or in Training (NEET) for both quantitative and qualitative components of the study, this sample was still over-represented by students and as such the interpretation of the results should take this into consideration. In addition, for both quantitative and qualitative aspects, recruitment of young adults at university/college was only possible through the institutions since direct access to students was not permitted. Major employers denied direct access to young adults in work places due to time constraints and data protection issues. Consequently, only a random sample from the community was possible in order to capture those at work. This highlights the recruitment issues in this age group, another potential limitation in generalising the results to those who work. In addition, it was impossible to calculate the response rate for the questionnaire survey in this study due to the institutional approach and subsequent lack of denominator. While acknowledging the limitation of representativeness of this sample despite diligent attempts, this is a large study in this hard to reach age group.

While there is an argument that tackling obesity is the responsibility of the individual, this alone is unlikely to solve the problem of obesity (Chesney, Thurston, & Thomas, 2001). Some interventions might lead to weight loss in some targeted motivated populations (Poobalan, Aucott, Precious, Crombie, & Smith, 2010). However, replicating these interventions at community level is unlikely to succeed since only a fraction of young adults actually participate and among those, few will lose weight. In addition such weight losses are often not sustainable in the current obesogenic environment. Individual responsibility can only be successful with access to healthy lifestyle options (WHO, 2011). This suggests government, private and voluntary sectors should work together to change the societal and environmental factors, whilst supporting individuals who want to make healthy choices (Huang et al., 2003; Swinburn, Gill, & Kumanyika, 2005; Yach, McKee, Lopez, & Novotny, 2005).

## 6. Conclusion

Young adults (18–25 year olds) are vulnerable to weight gain but are difficult to reach. Elements deemed important by this specific group of young adults have been identified by this mixed method study and will inform development of an intervention. They need encouragement to have regular meals, less snacking, and require support to promote healthy diets, such as improved cooking skills. It is also important to consider factors such as their appearance, the need for variety of food and skills, and to account for eating in healthy and unhealthy phases. A targeted approach has been indicated in this study and might be a starting point in order to promote healthy living and in turn prevent obesity in this vulnerable age group.
